# Encoding Conditions Affect Recognition of Vocally Expressed Emotions Across Cultures

**DOI:** 10.3389/fpsyg.2013.00111

**Published:** 2013-03-13

**Authors:** Rebecca Jürgens, Matthis Drolet, Ralph Pirow, Elisabeth Scheiner, Julia Fischer

**Affiliations:** ^1^Cognitive Ethology Laboratory, German Primate CenterGöttingen, Germany

**Keywords:** acoustics, culture, emotion, play-acting, recognition, speech, vocalization

## Abstract

Although the expression of emotions in humans is considered to be largely universal, cultural effects contribute to both emotion expression and recognition. To disentangle the interplay between these factors, play-acted and authentic (non-instructed) vocal expressions of emotions were used, on the assumption that cultural effects may contribute differentially to the recognition of staged and spontaneous emotions. Speech tokens depicting four emotions (anger, sadness, joy, fear) were obtained from German radio archives and re-enacted by professional actors, and presented to 120 participants from Germany, Romania, and Indonesia. Participants in all three countries were poor at distinguishing between play-acted and spontaneous emotional utterances (58.73% correct on average with only marginal cultural differences). Nevertheless, authenticity influenced emotion recognition: across cultures, anger was recognized more accurately when play-acted (*z* = 15.06, *p* < 0.001) and sadness when authentic (*z* = 6.63, *p* < 0.001), replicating previous findings from German populations. German subjects revealed a slight advantage in recognizing emotions, indicating a moderate in-group advantage. There was no difference between Romanian and Indonesian subjects in the overall emotion recognition. Differential cultural effects became particularly apparent in terms of differential biases in emotion attribution. While all participants labeled play-acted expressions as anger more frequently than expected, German participants exhibited a further bias toward choosing anger for spontaneous stimuli. In contrast to the German sample, Romanian and Indonesian participants were biased toward choosing sadness. These results support the view that emotion recognition rests on a complex interaction of human universals and cultural specificities. Whether and in which way the observed biases are linked to cultural differences in self-construal remains an issue for further investigation.

## Introduction

Emotions are an important part of human social life. They mediate between the internal state and external world and they prepare the organism for subsequent actions and interactions. Although there is an ongoing debate about the definition of emotions (see for example Mason and Capitanio, 2012; Mulligan and Scherer, 2012; Scarantino, 2012), there is a growing consensus among theorists that emotion needs to be viewed as a multi-component phenomenon (Scherer, 1984; Frijda, 1986; Lazarus, 1991). The three major components of emotions are neurophysiological response patterns in the central and autonomic nervous systems; motor expression in face, voice, and gesture; and subjective feelings. Many theorists also include the evaluation or appraisal of the antecedent event and the action tendencies generated by the emotion as additional components of the emotional process (Scherer, 1984; Smith and Ellsworth, 1985; Frijda, 1986; Lazarus, 1991).

Different theoretical frameworks have been put forward as to whether emotions are universal and evolved adaptations (Darwin, 1872) or whether they are socially constructed and vary across cultures (Averill, 1980). Both approaches are, however, not mutually exclusive, and it has recently been argued that the dichotomy between nature and nurture should be abandoned (Prinz, 2004; Juslin, 2012; Mason and Capitanio, 2012). Matsumoto (1989), for example, argued that although emotions are biologically programed, cultural factors have a strong influence on the control of emotional expression and perception.

Scherer and Wallbott (1994) conducted a series of cross-cultural questionnaire studies in 37 countries to investigate the influence of culture on the experience of emotions and found strong evidence for both universality and cultural specificity in emotional experience, including both psychological and physiological responses to emotions. Ekman and colleagues (Ekman et al., 1969; Ekman and Friesen, 1971; Ekman and Oster, 1979) tested the universality of facial expressions and demonstrated that a standardized set of photographs depicting different emotion expressions was correctly judged by members of different, partly preliterate, cultures. At the same time, recognition accuracy was higher for members of the cultural background from which the facial expressions were obtained. Thus, facial expressions are considered to be largely universal (but, see Jack et al., 2012), while cultural differences are observed in the types of situations that elicit emotions (Matsumoto and Hwang, 2011), in small dialectic-like differences (Elfenbein et al., 2007), and in the culture-specific display rules that alter facial expressions (Ekman and Friesen, 1969; Matsumoto et al., 2008).

The human voice is also an important modality in the transmission of emotional information, both through verbal and non-verbal utterances (Banse and Scherer, 1996; Juslin and Laukka, 2003; Hammerschmidt and Jürgens, 2007; Sauter et al., 2010). Expression of emotion in the voice occurs via modifications of voice quality (Gobl and Ni Chasaide, 2003) and prosody in general (Scherer, 1986). Initial research on vocal emotion recognition indicated that the patterns in prosodic recognition were largely universal (Frick, 1985), which paralleled the results from facial expressions (Elfenbein and Ambady, 2002). Ratings of vocalizations by listeners showed that they were able to infer vocally expressed emotions at rates higher than chance (Banse and Scherer, 1996; Juslin and Laukka, 2003). In a classic study, Scherer et al. (2001) compared judgments by Germans and members of eight other cultures on expressions of emotions by German actors. They found that with increasing geographical distance from the speakers the recognition accuracy for emotional expressions decreased. Additionally, recognition accuracy was greater for foreign judges whose own language was closer to the Germanic language family. A meta-analysis on emotion recognition within and across cultures revealed that the in-group advantage found by Scherer et al. (2001) for German judges is a typical finding in cross-cultural emotion recognition studies (Elfenbein and Ambady, 2002). This meta-analysis included studies that used different types of stimuli, from facial and whole-body photographs to voice samples and video clips. Emotions were universally recognized at better-than-chance levels. However, there was also a consistent in-group advantage: accuracy was higher when emotions were both expressed and recognized by members of the same national, ethnic, or regional group. This advantage was smaller for cultural groups with greater exposure to one another, measured in terms of living in the same nation, physical proximity, and telephone communication (Elfenbein and Ambady, 2002).

Cultural variations in emotion recognition can not only be explained by differences in the emotion encoding, but also by response biases on part of the recipient due to culture-dependent decoding rules (Matsumoto, 1989; Elfenbein et al., 2002). For example, revealing that Japanese participants were less accurate in recognizing anger, fear, disgust, and sadness, Matsumoto (1992) suggested a bias against negative emotions in collectivistic societies as an important factor to maintain group stability (but, see Elfenbein et al., 2002 for divergent results).

Much of the research cited above has been performed on stereotypical and controlled expressions of emotions often produced by actors. Though actors spend many years perfecting the authenticity and clarity of their portrayals of human behavior and emotions (Goldstein and Bloom, 2011), acted emotional expressions may still be more stereotyped and more intense than spontaneous expressions (Wilting et al., 2006; Laukka et al., 2012, but, see Jürgens et al., 2011; Scherer, 2013), and are thought to be more strongly bound by social codes (Hunt, 1941; Matsumoto et al., 2009). In addition, preselected, stereotypical expressions might conceal possible effects of response biases in cross-culture studies due to their clear and unmistakable expression patterns (Wagner, 1993; Elfenbein et al., 2002).

In a series of previous studies we presented listeners with emotional speech tokens produced without external instruction (“authentic”) obtained from radio archives, as well as corresponding tokens re-enacted by professional actors (“play-acted”). We found that (German) listeners were poor at distinguishing between authentic and play-acted emotions. Intriguingly, the recording conditions nevertheless had a significant effect on emotion recognition. Anger was recognized best when play-acted, while sadness was recognized best when authentic (Drolet et al., 2012). Moreover, using an fMRI approach, we found that both explicit recognition of the source of the recording, i.e., whether it was authentic or play-acted (compared to the recognition of emotion) and authentic stimuli (versus play-acted) lead to an up-regulation in the ToM network (medial prefrontal, retrosplenial, and temporoparietal cortices). Moreover, acoustic analyses revealed significant differences in the F0 contour, with a higher variability in F0 modulation in play-acted than authentic stimuli (Jürgens et al., 2011).

Based on these findings, we here aim to expand our understanding of the recognition of play-acted and authentic stimuli and biases in emotion recognition. By testing participants from different cultures we intended to gain insights into the influence culture has on our findings. We selected Romanian and Indonesian participants because they differ in terms of the distance to the German sample, with a higher degree of overlap between the Romanian and German cultures than between Indonesian and German. Moreover, Romania and Indonesia have been described as collectivistic societies in contrast to the individualistic German society (Hofstede, 1980, 1996; Trimbitas et al., 2007), which allows a comparison of listeners’ culture-dependent response biases on non-instructed, more ambivalent speech tokens (Matsumoto, 1992; Elfenbein et al., 2002). If the observed interaction between emotion recognition and recording condition is based on universal processes in emotion recognition, we would predict a similar pattern across the three cultures. Specifically, more stereotyped displays should be recognized more easily across cultures (Elfenbein et al., 2007). If, in contrast, acting reflects a socially learned code, then the higher recognition of play-acted anger should disappear in the other two cultures (Hunt, 1941; Matsumoto et al., 2009), with a stronger effect in Indonesian than Romanian participants, due to cultural distance. If collectivistic societies foster a response bias against negative emotions, Romanian and Indonesian participants should reveal a bias against judging an emotion as anger, fear, or sadness in contrast to the German participants (Matsumoto, 1992; Elfenbein et al., 2002). This effect should be increased in cases in which the stimulus material is less clear and less stereotypical (Wagner, 1993; Elfenbein et al., 2002).

## Materials and Methods

### Recordings

We focused on four emotions that differ in terms of valence, dominance, and intensity: anger, fear, joy, and sadness (de Vignemont and Singer, 2006; Bryant and Barrett, 2008; Ethofer et al., 2009). These are the most commonly used emotions in this field of research (Sobin and Alpert, 1999; Scherer et al., 2001; Juslin and Laukka, 2003) and were accessible in the radio interviews used for stimulus material. Neutral prosody, while interesting for comparative reasons, is rare and hard to control in real-life settings. One possibility, news anchors, whose voices are characterized by neutral prosody, unfortunately represent a way of speaking more related to acting than to natural speech. We compared emotional expressions that were obtained during radio interviews to re-enacted versions of the same stimuli. The authentic speech recordings were selected from the database of a radio station and consisted of German expressions of fear, anger, joy, or sadness. The recordings were made during interviews with individuals talking in an emotional fashion about a highly charged ongoing or recollected event (e.g., parents speaking about the death of their children, people winning in a lottery, being in rage about current or past injustice, or threatened by a current danger). Emotions were ascertained through the content of the text spoken by the individuals, as well as the context. While the possibility of social acting can never be completely excluded we aimed to minimize this effect by excluding clearly staged settings (e.g., talk-shows). Stimuli were saved in wave format with 44.1 kHz sample rate and 16 bit sampling depth. Only recordings of good quality and low background noise were selected. Prior to the experiment, we asked 64 naïve participants to rate the transcripts for emotional content to ensure that the stimulus material was free of verbal content that could reveal the emotion. Text segments that were assigned to a particular emotion above chance level were shortened or deleted from the stimulus set. Thus, the stimuli that were used in the experiment did not contain any keywords that could allow inference of the expressed emotion, as for example: “I have known him for 43 years” (translation; original German: “Ich kenn ihn 43 Jahr”) was used as a sad stimulus, and “up to the window crossbar” (German: “bis zum Fensterkreuz”) as a fear stimulus. Of the chosen 80 speech tokens, 35 were made outdoors and varied in their noise surroundings. The final stimulus set consisted of 20 samples of joy and sadness, 22 samples of anger, and 18 samples of fear, half of which were recorded from female speakers, resulting in a total of 80 recordings made by 78 different speakers. Segments had a mean length of 1.9 s (SD: 1.2 s). These wave files represent the so-called authentic stimuli. An information sheet was prepared for each authentic stimulus, which indicated the gender of the speaker, the context of the situation described, and a transliteration of the spoken text surrounding and including the respective selection of text.

The play-acted stimuli were produced by 21 male and 21 female actors (incl. 31 professional actors, 10 drama students, and 1 professional singer) recruited in Berlin, Hanover, and Göttingen, Germany. Actors were asked to reproduce two to three of the authentic recordings. Using the recording information sheet, the actors were told to express the respective text and emotion in their own way, using only the text, identified context, and emotion (the segment to be used as stimulus was not indicated and the actors never heard the original recording). Each actor could practice as long as needed, could repeat the acted reproduction as often as they required, and the recording selected for experimental use was the repetition each actor denoted as their first choice. To reduce any category effects between authentic and play-acted stimuli, the environment for the play-acted recordings was varied and 30 out of 80 randomly selected re-enactments were recorded outside. Nevertheless, care was taken to avoid excessive background noise. The relevant play-acted recordings (wave format, 44.1 kHz, 16 bit sampling depth) were then edited so they contained the same segment of spoken text as the authentic recordings. The average amplitude of all stimuli was equalized with AvisoftSASLab Pro Recorder v4.40 (Avisoft Bioacoustics, Berlin, Germany).

### Ethics

It was not possible to obtain informed consent from the people whose radio statements were used, as these were not individually identified. The brevity of the speech samples also precluded individual identification; we thus deemed the use of these samples as ethically acceptable. Actors gave verbal informed consent and were paid €20; experimental participants gave written informed consent and were paid €5 for their participation. Both actors and participants were informed afterward about the purpose of the study.

### Procedure

Due to the unequal numbers of speakers in the two conditions, we split the dataset in two and presented the two sets (playback A and playback B) to different groups of listeners. This also served to avoid participant exhaustion. Each set contained five authentic and five corresponding play-acted duplicates per speaker gender and intended emotion, resulting in a total of 80 stimuli (40 authentic, 40 play-acted) per set. Apart from three exceptions the playbacks were prepared in such a way that each actor was present in one set only once and related recordings (authentic versus play-acted) were presented in a pseudo-randomized fashion with the stipulation that speech token pairs were not played immediately after each another to make direct comparisons between recording pairs unlikely.

Each of the two sets of stimuli was presented to 20 listeners (10 female and 10 male) per country, resulting in 40 participants per country. In Germany, all participants were native German speakers recruited at the Georg-August University, Göttingen. Thirty-six were students, three were Ph.D. students, and one was an assistant lecturer. The age of German listeners varied between 20 and 33 years, the average age was *M* = 24.4, SD = 2.8 years for the listeners of playback A and *M* = 25.1, SD = 3.0 years for the listeners of playback B. The 40 Romanian listeners were recruited at the Lucian-Blaga-University of Sibiu, Romania. All of them were students. The age of Romanian listeners varied between 18 and 22 years, the mean age was *M* = 20.0, SD = 1.2 years for the listeners of playback A and *M* = 19.5, SD = 0.7 years for the listeners of playback B. The 40 Indonesian listeners were recruited at the Jakarta University, Indonesia. All Indonesian participants were students aged 18–31 years. The mean age was *M* = 20.7, SD = 2.8 years for the listeners of playback A and *M* = 20.5, SD = 1.9 years for the listeners of playback B. Neither the Romanian nor the Indonesian participants spoke any German. Romanian participants were, however, more familiar with German due to a large German community in the town of Sibiu. We did not collect any information about the emotional state of the participants before or during the experiments.

The stimuli were played back using a laptop (Toshiba Satellite with a Realtek AC97 Soundcard) via a program called Emosurvey (developed by Martin Schmeisser). Participants heard the stimuli via earphones (Sennheiser HD 497). They could activate the playback of the stimuli themselves and each stimulus could be activated a maximum of three times. The ratings were made via mouse clicks on the screen. When all questions were answered, the next stimulus could be activated. The listeners’ ratings were automatically saved in a log file, which could afterward be transferred to other software packages for analysis. In a forced-choice design participants were asked to determine, for each stimulus, the emotion expressed (emotion rating: joy, fear, anger, sadness), and whether the emotion was authentic or play-acted (dichotomous authenticity rating: authentic, play-acted).

### Statistical analysis

All models were implemented in the R statistical computing environment (R Developmental Core Team, 2008). We analyzed the authenticity ratings as well as the emotion ratings with generalized linear mixed models (GLMM) using the glmer function from the lme4 package for binomial data (Bates, 2005). The responses for correct authenticity rating and for correct emotion rating were tested with the predictor variables Country, Intended emotion, Stimulus authenticity, as well as their interactions, and the random factors Participant and Text stimulus (model formulation: correct recognition ∼ Country × Emotion × Authenticity + Random factor Text stimulus + Random factor Participant). Both models (Authenticity rating and Emotion rating) were compared to their respective null models (including only the intercept and the random factors, model formulation: correct recognition ∼ 1 + Random factor Text stimulus + Random factor Participant) using a likelihood ratio test (function ANOVA with the test argument “Chisq”). This comparison revealed differences, such that each of the full models accounted for more variance than the null models. Based on the chosen model we specified a set of experimental hypotheses that we tested *post hoc* using the glht function from the multcomp package (Hothorn et al., 2008), adjusting the *p*-values for multiple testing via single-step method.

Assessing recognition accuracy by simply counting hit rates, without addressing potential false alarms or biases (a strong preference toward one response), can be misleading (Wagner, 1993). For instance, if participants have a strong preference for rating stimuli as “authentic,” then one would obtain high hit rates for “authentic” speech tokens, but also many wrongly classified play-acted ones (called false alarms). Although the mean recognition rate in this case is quite high, the true ability to recognize authenticity is low. This example shows the importance of calculating biases for understanding rating behavior. A standardized method for analyzing the true discrimination ability for two response options was first introduced as Signal Detection Theory (SDT; Tanner et al., 1954). This technique offers both a measure of discriminatory ability *d*′ (also called sensitivity) which is the true ability to discern one stimulus from another, and a measure of the response bias toward one category, which is independent of sensitivity (criterion c). As the emotion recognition task in our study included four response options (four emotions), we analyzed the ratings using Choice Theory (Luce, 1959, 1963; Smith, 1982). Choice theory is a logit-model analog to SDT, which allows the analysis of more than two discrete response categories. A Choice Theory analysis provides (1) the participants’ relative bias (*b*), which is the equivalent criterion c and (2) dissimilarity values (α), which are equivalent to the discriminatory ability *d*′.

We implemented the choice theory analysis as a baseline-category logit-model (Agresti, 2007). We used the fitted intercept and slope coefficients to derive the bias and similarity parameters of choice theory. The binomial “mixed” model for authenticity recognition (binomial due to the two response options “authentic” and “play-acted”) was calculated in R using the glmer function of the lme4 package (Bates, 2005). The multinomial “mixed” model for emotion recognition was programed under WinBUGS (Lunn et al., 2000) using the R2WinBUGS interface package (Sturtz et al., 2005) to account for the four response options (“anger,” “fear,” “sadness,” and “joy”).

## Results

### Authenticity recognition

**Figure 1:**
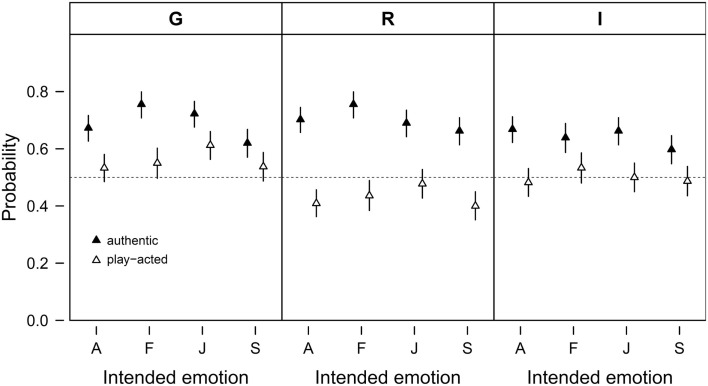
**Probability of correct authenticity recognition by intended emotion (A – anger, F – fear, J – joy, S – sadness) and stimulus authenticity (authentic or play-acted)**. The data are split by cultural affiliation (G – Germany, R – Romania, I – Indonesia). Given are means and 95% confidence intervals. The probability of correct authenticity recognition by chance is 0.5 as indicated by the dashed horizontal lines.

Across cultures, recognition accuracy for authenticity was only slightly above chance (*M* = 58.73%, SD = 8.84%), with a higher recognition rate for authentic (*M* = 67.81%, SD = 12.37) than for play-acted speech tokens (*M* = 49.58%, SD = 16.78). *Post hoc* tests confirmed this difference in recognition rates (*z* = 18.39, *p* < 0.001; Figure 1). German raters, correct in 62.43% of cases, were, on average, more accurate in their authenticity ratings than either Romanian (57.20%) or Indonesian raters (56.67%; German – Romanian *z* = 2.99, *p* = 0.028; German – Indonesian *z* = 2.95, *p* = 0.031).

**Figure 2:**
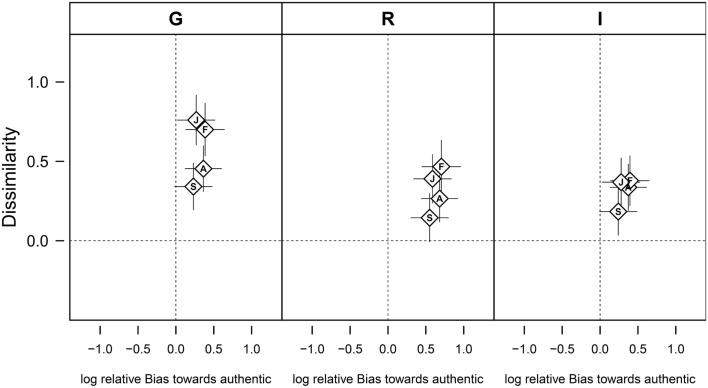
**Discrimination of authentic and play-acted vocal expressions of emotions as assessed by choice theory**. The discriminatory ability is described by the dissimilarity between authentic and play-acted stimuli (depicting how well the stimuli could be discriminated) and by the participants’ relative bias toward choosing authentic as a response, which are plotted against each other. The figure shows how these parameters vary in dependence of cultural affiliation (G – Germany, R – Romania, I – Indonesia) and the intended emotional content (A – anger, F – fear, J – joy, S – sadness). Positive values on the *x*-axis indicate a bias toward preferentially choosing the response “authentic,” while higher dissimilarity values indicates a better ability to distinguish the stimuli. Data are given are as means ± 95% confidence intervals.

The analysis of ratings using choice theory revealed that participants had a strong bias toward choosing the response “authentic” in the authenticity ratings (Figure 2), which may explain the higher recognition accuracy for authentic speech tokens. The *post hoc* pair-wise comparisons between the participants of the different countries revealed a significantly greater bias in Romanians than Germans (*z* = 2.64, *p* = 0.045; Figure 2).

The overall mean dissimilarity of 0.40 implies a generally low discriminatory capability between authentic and play-acted vocal expressions of emotions (MacMillan and Creelman, 2005). *Post hoc* tests revealed that German participants had a higher dissimilarity value and thus a better discriminatory ability than Romanian and Indonesian participants (German-Romanian: *z* = 4.535, *p* < 0.001; German – Indonesian: *z* = 4.590, *p* < 0.001).

### Emotion recognition

**Figure 3:**
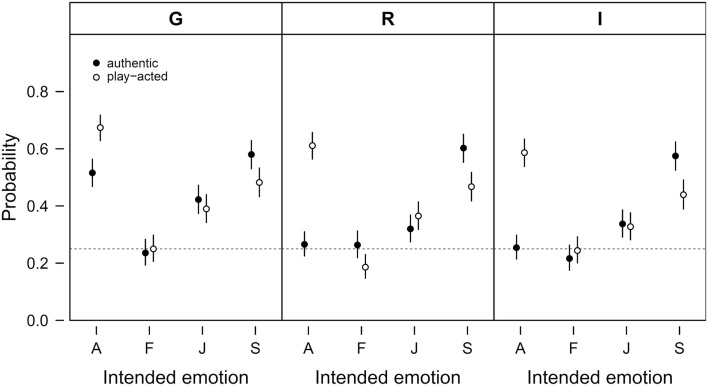
**Probability of correct emotion recognition**. Given is the probability of correct emotion recognition with respect to the intended emotion (A – anger, F – fear, J – joy, S – sadness) and stimulus authenticity (authentic or play-acted). The data are split by cultural affiliation (G – Germany, R – Romania, I – Indonesia). Given are means and 95% confidence intervals. The probability of correct emotion recognition by chance is 0.25 as indicated by the dashed horizontal lines.

In total, the correct response rate in emotion ratings was 40.65% (SD = 6.41%), which is higher than a chance response rate of 25% resulting from a random selection of one of the four emotions. The emotion recognition ratings in general showed similar patterns in the three countries (Figure 3). The GLMM analysis revealed that the rate of correct emotion recognition was influenced by Intended emotion, Stimulus authenticity, and Country (see Table 1 for the results of the *post hoc* analysis). Play-acted stimuli were recognized more accurately (42.78%) than authentic stimuli (38.52%). Specifically, play-acted anger was recognized more frequently than authentic anger and authentic sadness more than play-acted sadness. Authenticity did not significantly influence the emotion recognition rates for fear and joy. Concerning the four emotion categories, anger and sadness were on average recognized significantly more frequently than fear and sadness was recognized more frequently than joy. Finally, emotion recognition rates were significantly higher for German participants in comparison to Romanian and Indonesian participants, but not for Romanian participants in comparison to Indonesian participants (Table 1).

**Table 1:** ***Post hoc* tests of cultural affiliation, and stimulus-specific factors (stimulus authenticity, intended emotion) on the probability of correct emotion recognition**.

Linear hypotheses	Estimate	Std. error	*z* Value	Pr (>|*z*|)
Auth–play == 0	−0.175602	0.046608	−3.768	0.00226**
Germany–Romania == 0	0.291267	0.059652	4.883	<0.001***
Germany–Indonesia == 0	0.351577	0.059665	−5.893	<0.001***
Romania–Indonesia == 0	0.06031	0.06036	0.999	0.97849
A–F == 0	1.22244	0.242372	5.044	<0.001***
A–J == 0	0.536029	0.233757	2.293	0.23193
A–S == 0	−0.193133	0.233599	−0.827	0.99434
J–F == 0	0.686411	0.247431	2.774	0.06781
S–F == 0	1.415573	0.247282	5.725	<0.001***
S–J == 0	0.729162	0.238845	3.053	0.02912*
Auth–play (A) == 0	−1.356013	0.090051	−15.058	<0.001***
Auth–play (F) == 0	0.077003	0.105342	0.731	0.99776
Auth–play (J) == 0	−0.007027	0.088324	−0.08	1
Auth–play (S) == 0	0.583629	0.088031	6.63	<0.001***

The p-values are adjusted for multiple testing. Auth – non-instructed; play – instructed; A – anger; F – fear; J – joy; S – sadness. *p < 0.05; **p < 0.01; ***p < 0.001.

**Figure 4:**
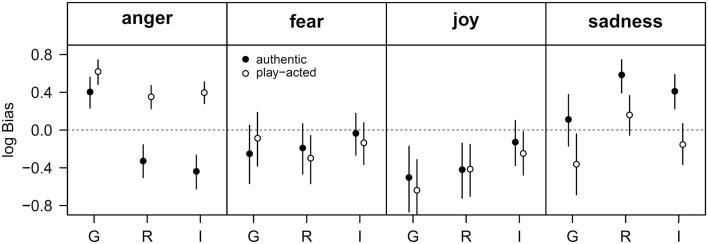
**Analysis of emotion recognition data by choice theory**. Given is the log-transformed response bias for each of the four possible choices (anger, fear, joy, sadness) with respect to cultural affiliation (G – Germany, R – Romania, I – Indonesia). The filled and open symbols indicate the response bias for authentic and play-acted stimuli. Data are given as means and 95% uncertainty interval. In the absence of any bias, all four log-transformed bias values would be zero. Positive values indicate a bias toward choosing the response named in the headline, whereas a value below zero indicates a bias against choosing the respective response.

The response bias for emotion judgments was calculated with respect to cultural affiliation and stimulus authenticity. In all three countries participants showed a bias toward rating play-acted stimuli as angry (Figure 4). This bias was higher for German than for Romanian or Indonesian participants. German participants were also biased toward rating authentic stimuli as angry, while Romanian and Indonesian participants preferentially chose “sadness” and were additionally biased against choosing “anger” when rating authentic stimuli. There was no effect of authenticity or country of origin with respect to the responses “joy” and “fear.” Indonesian participants, whose bias against “joy” was less distinct than for Romanian or German participants, were the only exception.

**Figure 5:**
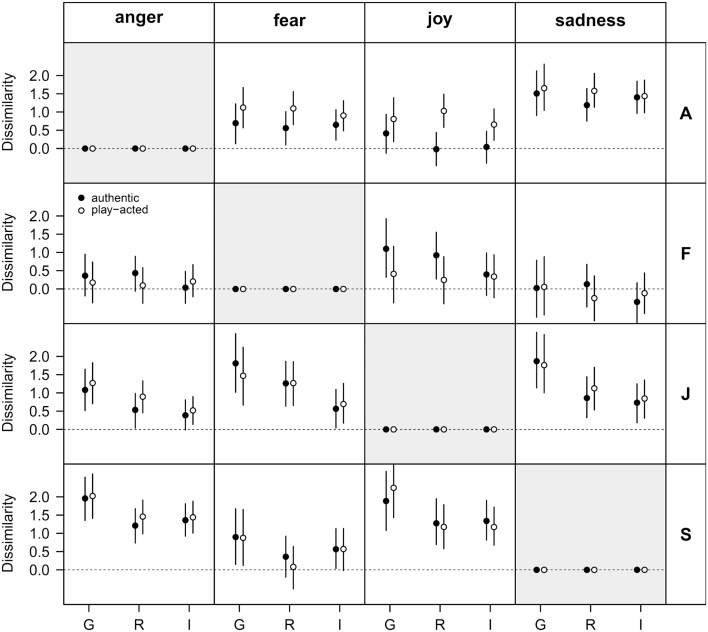
**Analysis of emotion recognition data using choice theory**. Given is the dissimilarity for different pairs of emotion stimuli with respect to cultural affiliation (G – Germany, R – Romania, I – Indonesia). The rows and columns of this matrix plot indicate the four emotion stimuli (A – anger, F – fear, J – joy, S – sadness) and the four possible responses (anger, fear, joy, sadness), respectively. Filled and open symbols refer to authentic and play-acted conditions, respectively. Data are given as means and 95% uncertainty interval. The dissimilarity describes how well each stimulus (depicted by rows) is discriminated from each other stimulus (depicted by response columns).

The outcome of the calculation of the dissimilarity values for all possible stimulus-response pairs during emotion ratings (including effects of country and stimulus authenticity) are shown in Figure 5. There were few differences between authentic and play-acted emotional expressions and between the participants of the three countries. High dissimilarity values were found between anger and sadness, which indicates that these emotions could be distinguished easily. The very low dissimilarity values for the stimulus “fear” (see row “F” in the matrix plot in Figure 5) indicate high confusion with the other emotion categories and reflect the low recognition rates for fear.

## Discussion

Participants in all three cultures had difficulties distinguishing between authentic (spontaneous) and play-acted (instructed) emotional expressions. The recognition of the expressed emotion also showed relatively low rates, but varied with respect to the emotion category and listener country of origin. Notably, the stimulus origin (authentic versus play-acted) had a clear impact on the recognition of vocal expressions of anger and sadness across all three cultures: anger was recognized more frequently when play-acted and sadness was recognized at higher rates when authentic, bolstering earlier findings for an independent German population (Drolet et al., 2012). While these results are significant, it remains unclear what leads to this effect. It may be that play-acted anger is more exaggerated than spontaneously expressed anger, while sadness, in contrast, is more difficult to play-act. On the other hand, it may be that, overall, some stimulus feature makes play-acted stimuli more likely to be perceived as anger and spontaneous stimuli as sadness.

With regard to our initial hypotheses, we found support for the conjecture that play-acted anger was recognized with higher accuracy than authentic anger across cultures, possibly because of its stereotypical nature. For the other three categories, acting does not necessarily appear to be connected with a more exaggerated expression, which is contrary to previous results (Barkhuysen et al., 2007; Laukka et al., 2012). According to our results, play-acted expressions do not represent a socially learned code (Matsumoto et al., 2009). Considering the similar interaction of emotion recognition and stimulus authenticity across the three cultures, our findings lend further support for the notion that emotion recognition is underpinned by human universals.

The fact that listeners of all three cultures were poor at discriminating between authentic and play-acted vocalizations shows that previous findings (Drolet et al., 2012) are applicable cross-culturally. If emotional expressions are indicators for underlying states that may require behavioral responses by the observer (see for controversial discussion, Russell et al., 2003; Barrett, 2011), the ability to detect fake emotional expressions should be important and evolutionarily adaptive (Schmidt and Cohn, 2001; Mehu and Scherer, 2012). The inability to distinguish between play-acted and spontaneous expressions is, therefore, counter-intuitive, but has also been found in previous studies (see for corresponding results, Ekman and O’Sullivan, 1991; Audibert et al., 2008). People tend to believe in the truthfulness of a statement rather than mistrust it (Zuckerman et al., 1984; Levine et al., 1999). This effect, labeled as “truth bias,” is reflected in our participants’ bias to choose the answer “authentic” when asked about the encoding condition of the emotional expression. It may be that the social cost of ignoring an emotion in others (miss) or wrongly considering others to be deceivers (false alarm) may make a bias toward believing in the authenticity of social signals advantageous (Ekman, 1996).

In addition to the well documented in-group effect for German participants (Scherer et al., 2001; Elfenbein and Ambady, 2002) in both emotion and authenticity recognition, cultural effects mainly became apparent in rating biases of emotions and not in recognition accuracy or dissimilarity. This has also been demonstrated by Sneddon et al. (2011), who showed that emotional stimuli were recognized similarly across different cultures, although the intensity ratings varied. Our initial hypothesis that Indonesian and Romanian participants exhibit a bias against negative emotions was, however, only partially supported. They had, in accordance to our hypothesis, a clear bias against selecting “anger,” but only for authentic stimuli. When listening to the spontaneous speech tokens, Indonesian and Romanian participants preferentially chose “sadness.” No cultural difference was found for the selection of “fear.” German participants showed a bias toward selecting “anger” for both authentic and play-acted stimuli. According to the hypothesis that individualistic cultures are expected to reinforce the expression of negative emotions, German participants may have expected a higher likelihood of being confronted with expressions of anger based on their everyday experiences, regardless of the stimulus type presented. Conversely, the more collectivistic Romanian and Indonesian participants may have expected expressions of sadness to be more likely (see Matsumoto, 1989 for similar results). Thus, sadness seems to rank differently compared to anger and the lumping of all negative emotions in the context of response bias seems to be an over-simplification, which might also explain the absence of clear bias effects in previous studies (Elfenbein et al., 2002; Sneddon et al., 2011). Interestingly, the expected response bias against “anger” for the Romanian and Indonesian participants is only present for authentic stimuli, which can be explained by stimulus-inherent features of the play-acted speech tokens overriding the response bias (Wagner, 1993; Elfenbein et al., 2002). The link between putative cultural biases requires stronger empirical investigations before firm conclusions can be drawn, in particular regarding limitations on the number and types of countries examined (with respect to language and cultural distance). However, our results demonstrate that the implicit effects of authenticity clearly derive from a complex interaction between stimulus-inherent features and cultural expectations about the likelihood of specific emotional expressions.

Due to the use of spontaneous emotional expressions taken from anonymous radio interviews, our study did not allow for a within-speaker design. We thus could not explicitly test whether individual differences in speaker expressivity affected the results. However, the large number of radio speakers and actors involved (more than generally seen in comparable studies) allowed us to minimize the influences of such effects. Additionally, the recognition rates of fear and joy were quite low compared to previous studies on vocal expressions of emotions (e.g., Van Bezooijen et al., 1983; Scherer et al., 2001; Pell and Kotz, 2011). This is interesting, taking into account that not only the spontaneous emotions, for which a low recognition would have been predicted, but also the play-acted ones, revealed recognition rates near chance levels. In contrast to standard methodology, we did not use exaggerated emotional expressions, preselected speech tokens, or emotional outbursts in a word or two (Van Bezooijen et al., 1983; Scherer et al., 2001; Pell et al., 2009). Actors were provided with longer transcripts (several sentences) to portray emotionally to ensure situations as similar to the authentic recordings as possible. It seems unlikely that specifically these professional actors were unable to encode joy or fear, considering that this has been done by laymen and inexperienced actors before (Van Bezooijen et al., 1983; Pell et al., 2009). In particular, the low recognition rates for joy and fear at or close to chance levels might reveal interesting facts about emotional expressions in general. The inability to recognize fear may indicate that fear is less clear in segments of longer speech samples than previously thought. In fact, we believe that the low recognition rates overall is what made the discovery of the interaction with authenticity, as well as the differences in the response bias, possible. It is clear that further work in this direction is needed to understand the relevance of emotion recognition research to day-to-day life. Nevertheless, the cross-cultural results revealed that spontaneous and play-acted emotional expressions are recognized similarly across cultures, indicating that both the recognition of play-acted and spontaneous emotional expressions rest on a similar universal basis. Furthermore, our results emphasize the importance of rating response biases, especially regarding more ambiguous expressions such as those taken from spontaneous situations.

## Conclusion

Combining all results, this study supports the view that emotion recognition rests on a complex interplay between human universals and cultural specificities. On the one hand, we found the same pattern of recognition and the same implicit effects of encoding conditions across cultures; on the other hand, cultural differences became evident in distinct biases. In addition, although the low recognition of encoding conditions would appear to argue for acted stimuli in vocal research, the implicit effects on emotion recognition seen here indicate that the design of future studies on vocal emotion recognition must take this variation in stimulus characteristics into account.

## Conflict of Interest Statement

The authors declare that the research was conducted in the absence of any commercial or financial relationships that could be construed as a potential conflict of interest.

## References

[B1] Agresti A. (2007). An Introduction to Categorical Data Analysis. New Jersey: Wiley

[B2] Audibert N. Aubergé V. Rilliard A. (2008). “How we are not equally competent for discriminating acted from spontaneous expressive speech,” in Proceedings of the Speech Prosody 2008, Campinas

[B3] Averill J. R. (1980). “A constructivist view of emotion,” in Emotion, Theory, Research and Experience, eds Plutchik R. Kellermann H. (New York: Academic Press), 305–339

[B4] Banse R. Scherer K. R. (1996). Acoustic profiles in vocal emotion expression. J. Pers. Soc. Psychol. 70, 614–636 10.1037/0022-3514.70.3.614 8851745

[B5] Barkhuysen P. Krahmer E. Swerts M. (2007). “Cross-modal perception of emotional speech,” in Proceedings of the International Congress of Phonetic Sciences (ICPhS’ 07), Saarbrücken

[B6] Barrett L. F. (2011). Was Darwin wrong about emotional expressions? Curr. Dir. Psychol. Sci. 20, 400–406 10.1177/0963721411422522

[B7] Bates D. (2005). Fitting linear mixed models in R using the lme4 package. R News 5, 27–30

[B8] Bryant G. A. Barrett H. C. (2008). Vocal emotion recognition across disparate cultures. J. Cogn. Cult. 8, 135–148 10.1163/156770908X289242

[B9] Darwin C. (1872). The Expression of Emotions in Man and Animal. London: John Murray

[B10] de Vignemont F. Singer T. (2006). The empathic brain: how, when and why? Trends Cogn. Sci. (Regul. Ed.) 10, 435–441 10.1016/j.tics.2006.08.008 16949331

[B11] Drolet M. Schubotz R. I. Fischer J. (2012). Authenticity affects the recognition of emotions in speech: behavioral and fMRI evidence. Cogn. Affect. Behav. Neurosci. 12, 140–150 10.3758/s13415-011-0069-3 22038706 PMC3267031

[B12] Ekman P. (1996). Why dont’ we catch liars? Soc. Res. 63, 801–817

[B13] Ekman P. Friesen W. V. (1969). The repertoire of nonverbal behavior: categories, origins, usage and coding. Semiotica 1, 49–98

[B14] Ekman P. Friesen W. V. (1971). Constants across cultures in face and emotion. J. Pers. Soc. Psychol. 17, 124–129 10.1037/h0030377 5542557

[B15] Ekman P. Oster H. (1979). Facial expressions of emotion. Annu. Rev. Psychol. 30, 527–554 10.1146/annurev.ps.30.020179.002523

[B16] Ekman P. O’Sullivan M. (1991). Who can catch a liar? Am. Psychol. 46, 913–920 10.1037/0003-066X.46.9.913 1958011

[B17] Ekman P. Sorenson E. R. Friesen W. V. (1969). Pan-cultural elements in facial displays of emotion. Science 164, 86–88 10.1126/science.164.3875.86 5773719

[B18] Elfenbein H. A. Ambady N. (2002). On the universality and cultural specificity of emotion recognition: a meta-analysis. Psychol. Bull. 128, 203–235 10.1037/0033-2909.128.2.203 11931516

[B19] Elfenbein H. A. Beaupre M. Levesque M. Hess U. (2007). Toward a dialect theory: cultural differences in the expression and recognition of posed facial expressions. Emotion 7, 131–146 10.1037/1528-3542.7.1.131 17352569

[B20] Elfenbein H. A. Mandal M. Ambady N. Harizuka S. Kumar S. (2002). Cross-cultural patterns in emotion recognition: highlighting design and analytical techniques. Emotion 2, 75–84 10.1037/1528-3542.2.1.75 12899367

[B21] Ethofer T. De Ville D. V. Scherer K. Vuilleumier P. (2009). Decoding of emotional information in voice-sensitive cortices. Curr. Biol. 19, 1028–1033 10.1016/j.cub.2009.10.030 19446457

[B22] Frick R. W. (1985). Communicating emotion – the role of prosodic features. Psychol. Bull. 97, 412–429 10.1037/0033-2909.97.3.412

[B23] Frijda N. H. (1986). The Emotions. Cambridge: Cambridge University Press

[B24] Gobl C. Ni Chasaide A. (2003). The role of voice quality in communicating emotion, mood and attitude. Speech Commun. 40, 189–212 10.1016/S0167-6393(02)00082-1

[B25] Goldstein T. R. Bloom P. (2011). The mind on stage: why cognitive scientists should study acting. Trends Cogn. Sci. (Regul. Ed.). 15, 141–142 10.1016/j.tics.2011.02.003 21398168

[B26] Hammerschmidt K. Jürgens U. (2007). Acoustical correlates of affective prosody. J. Voice 21, 531–540 10.1016/j.jvoice.2006.03.002 16647247

[B27] Hofstede G. (1980). Cultures’ Consequences. Berverly Hills, CA: Sage

[B28] Hofstede G. (1996). “The Nation-state as a source of common mental programming: similarities and differences across Eastern and Western Europe,” in The Future of Nation State – Essays on Cultural Pluralism and Political Integration, eds Gustavsson S. Lewin L. (London: Routledge), 2–20

[B29] Hothorn T. Bretz F. Westfall P. (2008). Simultaneous inference in general parametric models. Biom. J. 50, 346–363 10.1002/bimj.200810425 18481363

[B30] Hunt W. (1941). Recent development in the field of emotions. Psychol. Bull. 38, 249–276 10.1037/h0054615

[B31] Jack R. E. Garrod O. G. Yu H. Caldara R. Schyns P. G. (2012). Facial expressions of emotion are not culturally universal. Proc. Natl. Acad. Sci. U.S.A. 109, 7241–7244 10.1073/pnas.1200155109 22509011 PMC3358835

[B32] Jürgens R. Hammerschmidt K. Fischer J. (2011). Authentic and play-acted vocal emotion expressions reveal acoustic differences. Front. Psychol. 2:180 10.3389/fpsyg.2011.00180 21847385 PMC3148714

[B33] Juslin P. N. (2012). Are musical emotions invariant across cultures? Emot. Rev. 4, 283–284 10.1177/1754073912439773

[B34] Juslin P. N. Laukka P. (2003). Communication of emotions in vocal expression and music performance: different channels, same code? Psychol. Bull. 129, 770–814 10.1037/0033-2909.129.5.770 12956543

[B35] Laukka P. Audibert N. Aubergé V. (2012). Exploring the determinants of the graded structure of vocal emotion expressions. Cogn. Emot. 26, 710–719 10.1080/02699931.2011.602047 21851327

[B36] Lazarus R. S. (1991). Emotion and Adaption. New York: Oxford University Press

[B37] Levine T. R. Park H. S. McCornack S. A. (1999). Accuracy in detecting truths and lies: documenting the “veracity effect.” Commun. Monogr. 66, 125–144 10.1080/03637759909376468

[B38] Luce R. D. (1959). Individual Choice Behavior. New York: Wiley

[B39] Luce R. D. (1963). A threshold theory for simple detection experiments. Psychol. Rev. 70, 61–79 10.1037/h0039723 13931437

[B40] Lunn D. J. Thomas A. Best N. Spiegelhalter D. (2000). WinBUGS – a Bayesian modelling framework: concepts, structure, and extensibility. Stat. Comput. 10, 325–337 10.1023/A:1008929526011

[B41] MacMillan N. A. Creelman C. D. (2005). Detection Theory: A User’s Guide. London: Lawrence Erlbaum Assoc Inc

[B42] Mason W. A. Capitanio J. P. (2012). Basic emotions: a reconstruction. Emot. Rev. 4, 238–244 10.1177/1754073912439788 PMC4840933 27110280

[B43] Matsumoto D. (1989). Cultural influences on the perception of emotion. J. Cross Cult. Psychol. 20, 92–105 10.1177/0022022189201006

[B44] Matsumoto D. (1992). American-Japanese cultural differences in the recognition of universal facial expressions. J. Cross Cult. Psychol. 23, 72–84 10.1177/0022022192231005

[B45] Matsumoto D. Hwang H. S. (2011). Culture and emotion: the integration of biological and cultural contributions. J. Cross Cult. Psychol. 43, 91–118 10.1177/0022022111420147

[B46] Matsumoto D. Olide A. Willingham B. (2009). Is there an ingroup advantage in recognizing spontaneously expressed emotions? J. Nonverbal Behav. 33, 181–191 10.1007/s10919-009-0068-z

[B47] Matsumoto D. Seung Hee Y. Fontaine J. (2008). Mapping expressive differences around the world: the relationship between emotional display rules and individualism versus collectivism. J. Cross Cult. Psychol. 39, 55–74 10.1177/0022022108315489

[B48] Mehu M. Scherer K. R. (2012). A psycho-ethological approach to social signal processing. Cogn. Process. 13(Suppl. 2), 397–414 10.1007/s10339-012-0435-2 22328016

[B49] Mulligan K. Scherer K. R. (2012). Towards a working definition of emotion. Emot. Rev. 4, 345–357 10.1177/1754073912445818

[B50] Pell M. D. Kotz S. A. (2011). On the time course of vocal emotion recognition. PLoS ONE 6:e27256 10.1371/journal.pone.0027256 22087275 PMC3210149

[B51] Pell M. D. Paulmann S. Dara C. Alasseri A. Kotz S. A. (2009). Factors in the recognition of vocally expressed emotions: a comparison of four languages. J. Phon. 37, 417–435 10.1016/j.wocn.2009.07.005

[B52] Prinz J. (2004). “Which emotions are basic?” in Emotion, Evolution, and Rationality, eds Evans D. Cruse P. (Oxford: Oxford University Press), 69–88

[B53] R Developmental Core Team. (2008). R: A Language and Environment for Statistical Computing. Vienna: R Foundation for Statistical Computing

[B54] Russell J. A. Bachorowski J.-A. Fernández-Dols J.-M. (2003). Facial and vocal expressions of emotion. Annu. Rev. Psychol. 54, 329–349 10.1146/annurev.psych.54.101601.145102 12415074

[B55] Sauter D. A. Eisner F. Ekman P. Scott S. K. (2010). Cross-cultural recognition of basic emotions through nonverbal emotional vocalizations. Proc. Natl. Acad. Sci. U.S.A. 107, 2408–2412 10.1073/pnas.0908239106 20133790 PMC2823868

[B56] Scarantino A. (2012). How to define emotions scientifically. Emot. Rev. 4, 358–368 10.1177/1754073912445810

[B57] Scherer K. R. (1984). “On the nature and function of emotion: a component process approach,” in Approaches to Emotion, eds Scherer K. R. Ekman P. (Hillsdale, NJ: Erlbaum), 293–318

[B58] Scherer K. R. (1986). Vocal affect expression: a review and model for future research. Psychol. Bull. 99, 143–165 10.1037/0033-2909.99.2.143 3515381

[B59] Scherer K. R. (2013). Vocal markers of emotion: comparing induction and acting elicitation. Comput. Speech Lang. 27, 40–58 10.1016/j.csl.2011.11.003

[B60] Scherer K. R. Banse R. Wallbott H. G. (2001). Emotion inferences from vocal expression correlate across languages and cultures. J. Cross Cult. Psychol. 32, 76–92 10.1177/0022022101032001009

[B61] Scherer K. R. Wallbott H. G. (1994). Evidence for universality and cultural variation of differential emotion response patterning. J. Pers. Soc. Psychol. 66, 310–328 10.1037/0022-3514.66.2.310 8195988

[B62] Schmidt K. L. Cohn J. F. (2001). Human facial expressions as adaptations: evolutionary questions in facial expression research. Am. J. Phys. Anthropol. 116, 3–24 10.1002/ajpa.20001.abs 11786989 PMC2238342

[B63] Smith C. A. Ellsworth P. C. (1985). Patterns of cognitive appraisal in emotion. J. Pers. Soc. Psychol. 48, 813–838 10.1037/0022-3514.48.4.813 3886875

[B64] Smith J. E. K. (1982). Recognition models evaluated: a commentary on Keren and Baggen. Percept. Psychophys. 31, 183–189 10.3758/BF03206219 7079099

[B65] Sneddon I. McKeown G. McRorie M. Vukicevic T. (2011). Cross-cultural patterns in dynamic ratings of positive and negative natural emotional behaviour. PLoS ONE 6:e14679 10.1371/journal.pone.0014679 21364739 PMC3041750

[B66] Sobin C. Alpert M. (1999). Emotion in speech: the acoustic attributes of fear, anger, sadness and joy. J. Psycholinguist. Res. 28, 347–365 10.1023/A:1023237014909 10380660

[B67] Sturtz S. Ligges U. Gelman A. (2005). R2WinBUGS: a package for running WinBUGS from R. J. Stat. Softw. 12, 1–16

[B68] Tanner J. Wilson P. Swets J. A. (1954). A decision-making theory of visual detection. Psychol. Rev. 61, 401–409 10.1037/h0058700 13215690

[B69] Trimbitas O. Lin Y. Clark K. D. (2007). Arta de a cere scuze in cultura romaneasca: use of apology in ethnic Romanian culture. Hum. Commun. 10, 401–420

[B70] Van Bezooijen R. Otto S. A. Heenan T. A. (1983). Recognition of vocal expressions of emotion: a three-nation study to identify universal characteristics. J. Cross Cult. Psychol. 14, 387–406 10.1177/0022002183014004001

[B71] Wagner H. L. (1993). On measuring performance in category judgment studies of nonverbal behavior. J. Nonverbal Behav. 17, 3–28 10.1007/BF00987007

[B72] Wilting J. Krahmer E. Swerts M. (2006). “Real vs. acted emotional speech,” in Proceeding of the Interspeech-2006, Pittsburgh, PA

[B73] Zuckerman M. Koestner R. Colella M. J. Alton A. O. (1984). Anchoring in the detection of deception and leakage. J. Pers. Soc. Psychol. 47, 301–311 10.1037/0022-3514.47.2.301

